# Down-regulated *miR-495* can target programmed cell death 10 in ankylosing spondylitis

**DOI:** 10.1186/s10020-020-00157-3

**Published:** 2020-05-25

**Authors:** Wen-Juan Ni, Xiao-Min Leng

**Affiliations:** 1 School of Basic Medicine, Gannan Medical University, Ganzhou, 341000 Jiangxi, People’s Republic of China; 2 The First Affiliated Hospital of XinXiang Medical University, 453100 WeiHui, Henan People’s Republic of China

**Keywords:** MiR-495, Methylation, PDCD10, Biomarker, Target, Ankylosing spondylitis

## Abstract

**Background:**

MicroRNAs (miRNAs) play crucial roles in regulating eukaryotic gene expression. Recent studies indicated that aberrantly expressed miRNAs are involved in the pathogenesis of ankylosing spondylitis (AS). Indeed, hsa-miR-495-3p (*miR-495*) has been reported as an anti-oncogene in different cancers. However, the role of miR-495 in AS is still unknown.

**Methods:**

In this study, quantitative real-time polymerase chain reaction (PCR) was used to detect the expression of *miR-495* in the peripheral blood mononuclear cells (PBMCs), whole blood, and serum of patients with AS. Bisulfite-specific PCR sequencing and methylated DNA immunoprecipitation were used to detect the methylation in the promoter region of *miR-495*. To determine the influence of *miR-495* expression on the target gene, programmed cell death 10 (*PDCD10*), dual luciferase reporter assays together with an adenoviral vector containing the *miR-495* locus were used. Receiver operating characteristic (ROC) curves were used to evaluate the efficacy of miR-495 as a diagnostic biomarker of AS. Gene Ontology, Kyoto Encyclopedia of Genes and Genomes pathway analysis, and western blotting were used to explore the potential role of *miR-495* in AS pathogenesis and the mechanism by which it facilitates AS pathogenesis.

**Results:**

*miR-495* is down-regulated and the promoter region of *miR-495* is highly methylated in AS. The expression of *miR-495* is negatively associated with *PDCD10* expression in both patients with AS and healthy controls. Further experiments showed that *PDCD10* can be targeted by *miR-495*. The ROC curves of *miR-495* suggested that it is a very specific and sensitive biomarker for AS diagnosis. Bioinformatics analysis and signal pathway studies indicated that *miR-495* can down-regulate β-catenin and transforming growth factor-β1.

**Conclusions:**

Our studies indicated that down-regulation of *miR-495* can be used as a potential molecular marker for the diagnosis and treatment of AS, thus providing new insights into the role of miRNAs in AS pathology.

## Background

Ankylosing spondylitis (AS) is a chronic autoimmune disease that can result in functional and structural impairments by affecting the sacroiliac joint and the axial skeleton (Braun and Sieper 2007; Danve and O'Dell 2015; Brown et al. 2016). The prevalence of AS is approximately 0.24% in Europe, 0.17% in Asia, 0.32% in North America, 0.10% in Latin America, and 0.07% in Africa (Dean et al. 2014). Multilevel complex interactions between genetic, epigenetic and environmental factors play important roles during AS development (Zhu et al. 2019). As a chronic disease, the onset of AS is usually early and affects more men than women (Feldtkeller et al. 2003). Due to the nature of AS symptoms, the lag time between symptom onset and diagnosis is 8 to 11 years. Although modern imaging techniques, anti-inflammatory agents, and physiotherapy approaches have been developed for diagnosing and treating AS, significant challenges still remain in the early diagnosis and treatment of AS (Braun and Sieper 2007; Danve and O'Dell 2015).

MicroRNAs (miRNAs) are functionally important for eukaryotic cells (Bartel 2004; Krol et al. 2010). miRNAs typically regulate gene expression at the post-transcriptional level by dynamically interacting with different mRNAs (Zhang et al. 2009; Ni and Leng 2015). Since aberrant miRNA expression reflects the initiation and progression of pathological conditions, the validation of specific miRNAs as biomarkers for diseases has become a critical milestone in diagnostics (Wang et al. 2016). Well-documented studies show that aberrant expression of miRNAs can contribute to the pathogenesis of AS (Lai et al. 2013; Li et al. 2016; Mohammadi et al. 2018). Previous studies of hsa-miR-495-3p (*miR-495*) indicated its importance in cellular development and differentiation (Clark et al. 2016; Li et al. 2017), and it has been functionally described as a tumor suppressor in multiple tumors (Ahmadi et al. 2017; Chen et al. 2017; Eun et al. 2018; Liu et al. 2019). However, the role of *miR-495* in the pathogenesis of AS and the mechanism by which it facilitates AS pathogenesis remain elusive.

Programmed cell death 10 (*PDCD10*) protein, also known as TF-cell apoptosis-related protein 15 (*TFAR-15*), is widely expressed in different human tissues (Wang et al. 1999). As the third gene locus related to cerebral cavernous malformations (CCMs), *PDCD10* is alternatively named *CCM3* (Bergametti et al. 2005). Sequence conservation and binding studies suggest that *PDCD10* biases to form heterodimers with the germinal center kinase III (GCKIII) subfamily (Ceccarelli et al. 2011). *PDCD10* is an essential and versatile signal transduction molecule under different physiological and pathological conditions (Huang and Zhao 2013); however, its role and its relationship with *miR-495* in AS are yet to be elucidated.

In this study, the expression of *miR-495* in both AS patients and healthy controls was quantified. A high level of methylation in the promoter region of *miR-495* resulted in the lower expression of this miRNA in AS. Moreover, this miRNA can target *PDCD10* via interacting with its 3′UTR. The receiver operating characteristic (ROC) curves indicated that *miR-495*, particularly from peripheral blood mononuclear cells (PBMCs), was a highly specific and sensitive biomarker for the diagnosis of AS. Our results showed that Wnt and TGF-β signal pathways, which play essential roles in AS pathology, can be down-regulated by *miR-495*. This study suggests that *miR-495* may have application in the diagnosis and treatment of AS and provides new insight on the role of miRNAs in AS pathogenesis.

## Materials and methods

### Study subjects

This study was approved by the local institutional review board and the ethics committee of the First Affiliated Hospital of Xinxiang Medical University, Xinxiang, Henan (no. 2015095).Written informed consent was obtained from all participants. Exclusion criteria included pregnancy, malignancies, other rheumatological and chronic diseases, and lack of written informed consent from the patients. This study was carried out according to the principles expressed in the Declaration of Helsinki. Patients with AS were diagnosed according to the 1984 New York Modified Criteria (van der Linden et al. 1984). All subjects underwent a comprehensive physical examination, clinical chemistry analysis, and medical history before enrollment. All participant information is included in Table 1.

**Table 1:** Characteristics of healthy controls (HC) and AS patients

Characteristics	AS (*N* = 150)	HC (*N* = 150)
Gender (Male/Female)	Male (*N* = 114) Female (*N* = 36)	Male (*N* = 114) Female (*N* = 36)
Age (years)	M: 34.0 ± 12.5 F: 32.5 ± 10.5	M: 35.5 ± 10.5 F: 33.5 ± 8.5
Disease duration (years), mean ± SD	5.5 ± 0.8	0
HLA-B27, mean ± SD (0~147)	161.0 ± 8.5	Normal
ESR (mm/h), mean ± SD (0~15 for Male; 0~20 for Female)	22.5 ± 11.9	Normal
CRP (mg/dL), mean ± SD (1~15)	0.82 ± 0.54	Normal
BASDAI, mean ± SD	4.43 ± 1.35	Normal
BASFI, mean ± SD	36.8 ± 19.6	Normal
mSASSS	13.0 ± 7.0	Normal
Suggestions for immunosuppressant drugs		
Steroids	4%	0
DMARDs	6%	0
NSAIDs	100%	0

AS, ankylosing spondylitis; HC, healthy control; SD, Standard Deviation; HLA-B27, Human Leukocyte Antigen (HLA) B27; ESR, Erythrocyte Sedimentation Rate; CRP, C Reactive Protein; BASDAI, Bath Ankylosing Spondylitis Disease Activity; BASFI, Bath Ankylosing Spondylitis Functionality Index; mSASSS, modified Stoke Ankylosing Spondylitis Spine Score; DMARDs, Disease-Modifying Anti-Rheumatic Drugs; NSAIDs, Non-Steroidal Anti-Inflammatory Drugs

### RNA extraction

Blood samples collected from the ward were centrifuged in Ficoll solution (TBDscience, Code No: LTS1077). Then the cells were recovered from the media interface layer and washed twice with 1× phosphate buffered saline (PBS; Gibco, Code No: 20012050). Finally, cells were disrupted by RNAiso Plus (Takara, Code No: 9108) and total RNA was extracted according to the protocol. Total whole blood RNA extraction was according to the protocol (Takara, Code No: 9112). Total serum RNA was extracted according to the protocol (Qiagen, Code No: 217184), with *cel-miR-39* as a reference gene.

### Quantitative real-time PCR (qPCR)

qPCR primers were synthesized by Shanghai Sangon Biotech. Detailed primer sequences are summarized in the Supplementary Materials (S1). qPCR were performed according to the MIQE guidelines (Bustin et al. 2009). The cDNA was synthesized with modification using PrimeScript™ RT reagent kit with gDNA eraser (Takara, Code No: RR047A) and used directly in the SYBR Green qPCR reactions (Takara, Code No: RR420A); qPCR was performed using QuantStudio™ Dx Real-Time Instrument (Applied Biosystems, Code No: 4479889).

### Bisulfite-specific PCR sequencing (BSP)

Bisulfite-specific PCR sequencing primers were synthesized by Shanghai Sangon Biotech. Detailed primer sequences are summarized in the Supplementary Materials (S2). Genomic DNA was extracted according to the manufacturer’s protocol (Takara, Code No: 9450). The BSP kit was obtained from TIANGEN Biotech (Code No: DP215). DNA samples with or without BSP treatments were amplified by PCR (Takara, Code No: RR02MA) and cloned into T-A plasmids (Takara, Code No: 3271) for sequencing. Four clones from each sample were sequenced.

### Methylated DNA Immunoprecipitation (MeDIP) ChIP qPCR

The Methylated DNA Immunoprecipitation (MeDIP) ChIP Kit was obtained from Abcam (Code No: ab117135). Forward and reverse primer sequences for the *miR-495*, *PDCD10*, and glyceraldehyde 3-phosphate dehydrogenase (*GADPH*) promoters are listed in the Supplementary Materials (S3). After shearing by sonication, genomic DNA was immunoprecipitated using the Me-DIP Kit according to the manufacturer’s instructions. The treated DNA was then analyzed by qPCR, as described in the previous paragraph.

### Dual luciferase reporter assay

*MiR-495* mimics and the corresponding mutants were synthesized by Genepharma (Shanghai, China). HEK293T cells were cultured in Dulbecco’s Modified Eagle Medium (DMEM) containing 10% fetal bovine serum and 1% streptomycin/penicillin in a humidified air atmosphere with 5% CO_2_ at 37 °C. Wild-type and mutated 3′UTRs of *PDCD10* were cloned into the psiCHECK™-2 vector (Promega, USA). All transfections were conducted with Lipo2000 (Invitrogen, USA). The dual luciferase assay kit was obtained from Beyotime Institute of Biotechnology (Jiangsu, China). The experimental procedures were according to the manufacturer’s instructions. All assays were run on Glomax® (Promega, USA).

### Over-expression of *miR-495*

The adenovirus (vector: pDC316-mCMV-EGFP) carrying *miR-495* was obtained from Genechem (Shanghai, China). All the procedures were according to the manufacturer’s protocol. The multiplicity of infection used was 200, and the infection time was 48 h. The infected cells were then harvested for further studies.

### Primary culture of PBMCs

All the procedures followed were as per a previous report, with some modifications (Katial et al. 1998). After isolation by Ficoll, PBMCs were washed and cultured in RPMI1640 containing 10% fetal bovine serum and 1% streptomycin/penicillin in a humidified air atmosphere with 5% CO_2_ at 37 °C. After 24 h, cells were infected with adenovirus, and after 48 h, washed by PBS, and collected for qPCR and western blotting.

### Western blot

A radioimmunoassay buffer-containing cocktail was added to the collected cells. The bicinchoninic acid (BCA) assay kit (Thermo Scientific, Code No: 23225) was used to measure the concentrations of protein. After SDS-PAGE (10%), proteins were transferred to PVDF membranes (Thermo Scientific, Code No: 88518) and then incubated overnight at 4 °C in 5% nonfat milk with primary antibodies for PDCD10 (Immunoway, YN0271), β-catenin (Beyotime, AC106), TGF-β1 (Abcam, ab92486) and GADPH (Boster, M00227–5). The next day, a secondary antibody (Boster, BA1041) was used to probe the membranes. The protein bands were detected with the ECL chemiluminescence system (AmershamPharmacia Biotech, USA) and the gray values were quantified with the software Quantity One.

### Bioinformatics analysis

Gene Ontology (GO) and Kyoto Encyclopedia of Genes and Genomes (KEGG) analysis were performed using online DAVID tools (Huang da et al. 2009a, 2009b). The significant GO and signaling pathways were based on the *P* value threshold of 0.01. Predicted targets showing Pearson correlation *r* < 0 and *P* value < 0.05 were considered as candidates, together with targets that were experimentally validated in published reports.

### Statistical analysis

Data were reported as mean + SEM. Data within each cluster were analyzed with non-parametric ANOVA using Dunn’s procedure. Differences between two clusters were analyzed by a nonparametric two-tailed Mann–Whitney *U*-test. The Pearson correlation coefficient was calculated to evaluate the association between *PDCD10* and *miR-495*. The ROC curves were used to evaluate the sensitivity and specificity of *miR-495*. All statistical analyses were performed using GraphPad Prism (Version 8.0, GraphPad Software, Inc). *P* values < 0.05 were considered to indicate statistically significant differences.

## Results

### *miR-495* is down-regulated in AS

**Fig. 1:**
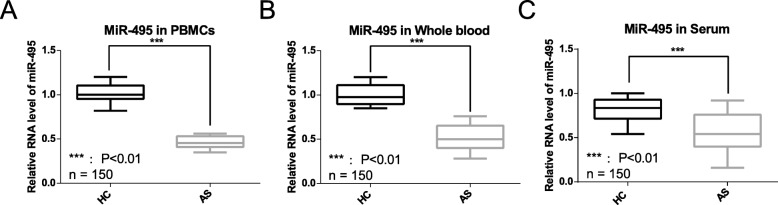
Detection of *miR-495* expression. The expression levels were presented as the mean + SD and calculated by 2^-△△ct^. **a** Expression of PBMC *miR-495*. *miR-495* expression in healthy controls and patients with AS is 1.02 + 0.11 and 0.464 + 0.071, respectively. **b** Expression of whole blood *miR-495*. *miR-495* expression in healthy controls and patients with AS is 0.993 + 0.116 and 0.523 + 0.158, respectively. **c** Expression of serum *miR-495*. *miR-495* expression in healthy controls and patients with AS is 0.813 + 0.147 and 0.36 + 0.162, respectively. AS: patients with ankylosing spondylitis. HC: healthy controls. *n* = 150: tested samples in each group. ***: *P* value < 0.01

*miR-495* has been reported to be an anti-oncogene in multiple cancers. To evaluate *miR-495* expression, we assessed its RNA level in different biological fluids in both patients with AS and healthy controls. Previous reports show that PBMCs, whole blood, and serum can act as a source of miRNAs to study AS (Lai et al. 2013; Li et al. 2016; Mohammadi et al. 2018). *miR-495* is significantly (*P* < 0.01) down-regulated in PBMCs from patients with AS (Fig. 1a). The down-regulation of *miR-495* was observed in both whole blood and serum (Fig. 1b and c).

### *miR-495* promoter is highly methylated

**Fig. 2:**
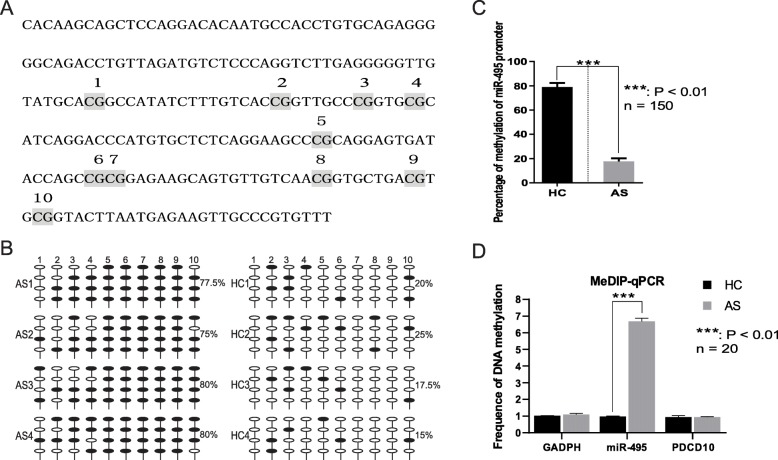
Methylation detection of *miR-495* promoter region. All data was presented as the mean + SD. **a** Sequence schema of the bisulfite-specific PCR sequenced region in the *miR-495* promoter region. Numbers (1–10) indicate potential CpG islands. **b** Methylation status of CpGs in the *miR-495* promoter region. White and black circles indicated un-methylated and methylated CpGs, respectively. AS1, AS2, AS3, AS4: different patients with AS. HC1, HC2, HC3, HC4: different healthy controls. **c** The percentage of *miR-495* promoter methylation. The percentage of *miR-495* promoter methylation in healthy controls and patients with AS was 0.79 + 3.30 and 0.18 + 2.63, respectively. **d** MeDIP-qPCR of *GADPH*, *miR-495*, and *PDCD10* promoters. The values of *GADPH* promoter methylation in healthy controls and patients with AS were 1.01 + 0.025 and 1.10 + 0.064, respectively. The values of *miR-495* promoter methylation in healthy controls and patients with AS were 0.97 + 0.036 and 6.68 + 0.224, respectively. The values of *PDCD10* promoter methylation in healthy controls and patients with AS were 0.93 + 0.11, and 0.94 + 0.028, respectively. *n* = 150 or 20: tested samples in each group. AS: patients with ankylosing spondylitis. HC: healthy controls. ***: *P* value < 0.01

To investigate the down-regulation of *miR-495* in AS, BSP was used to examine the *miR-495* promoter region. Sequences 2000 nucleotides upstream and 500 nucleotides downstream of mature *miR-495* were obtained and identified as the promoter region of *miR-495*. CpG islands of the sequences were analyzed by CpG island Searcher (http://cpgislands.usc.edu/) (Takai and Jones 2003). In the promoter region of *miR-495*, 10 potential CpG islands were present (Fig. 2a). Following BSP, we determined that the *miR-495* promoter is highly methylated in patients with AS (Fig. 2b), with nearly 80% of the nucleotides methylated, while only about 20% were methylated in healthy controls (Fig. 2c). MeDIP ChIP qPCR also confirmed that miR-495 promoter region is highly methylated in patients with AS (Fig. 2d).

### *miR-495* is negatively associated with *PDCD10*

**Fig. 3:**
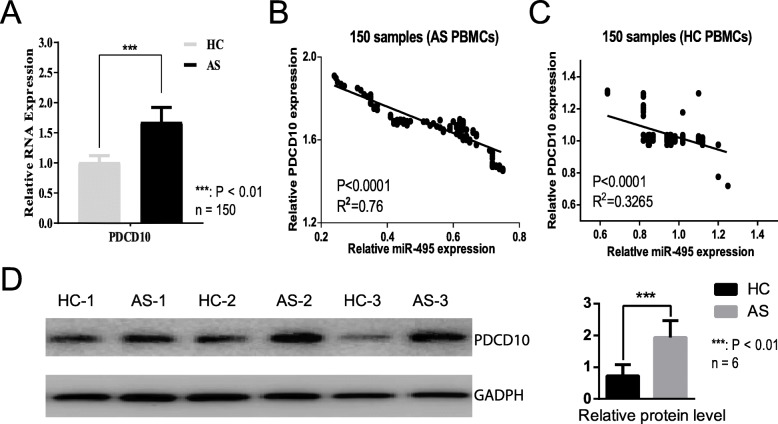
Expression of *PDCD10*. **a** qPCR of *PDCD10*. The expression levels were presented as the mean + SD and calculated by 2^-△△ct^. *PDCD10* expression in healthy controls and patients with AS was 1.01 + 0.116 and 1.68 + 0.241, respectively. **b** Correlation analysis between *PDCD10* and *miR-495* expression in patients with AS. **c** Correlation analysis between *PDCD10* and *miR-495* expression in healthy controls. **d** Western blot of *PDCD10*. HC-1, HC-2, HC-3: different healthy controls. AS-1, AS-2, AS-3: different patients with AS. *GADPH* used as internal reference. The *PDCD10* protein levels were also showed as the histogram. *n* = 150 or 6: tested samples in each group. PBMCs: peripheral blood mononuclear cells. AS: patients with ankylosing spondylitis. HC: healthy controls. ***: *P* value < 0.01

As a potentially functional molecule in AS, it is unclear whether *miR-495* can target novel mRNAs. After a thorough analysis of StarBase, miRbase and other databases, we chose *PDCD10* for further studies (Griffiths-Jones et al. 2006; Yang et al. 2011). Expression of *PDCD10* is up-regulated in AS (Fig. 3a) and its mRNA level is negatively associated with *miR-495* in both patients with AS and healthy controls (Fig. 3b and c). In addition to its RNA, western blots confirmed that *PDCD10* protein levels increased in AS (Fig. 3d). These results indicated that *PDCD10* is up-regulated in AS, acting as a potential target of *miR-495*.

### miR-495 can target PDCD10

**Fig. 4:**
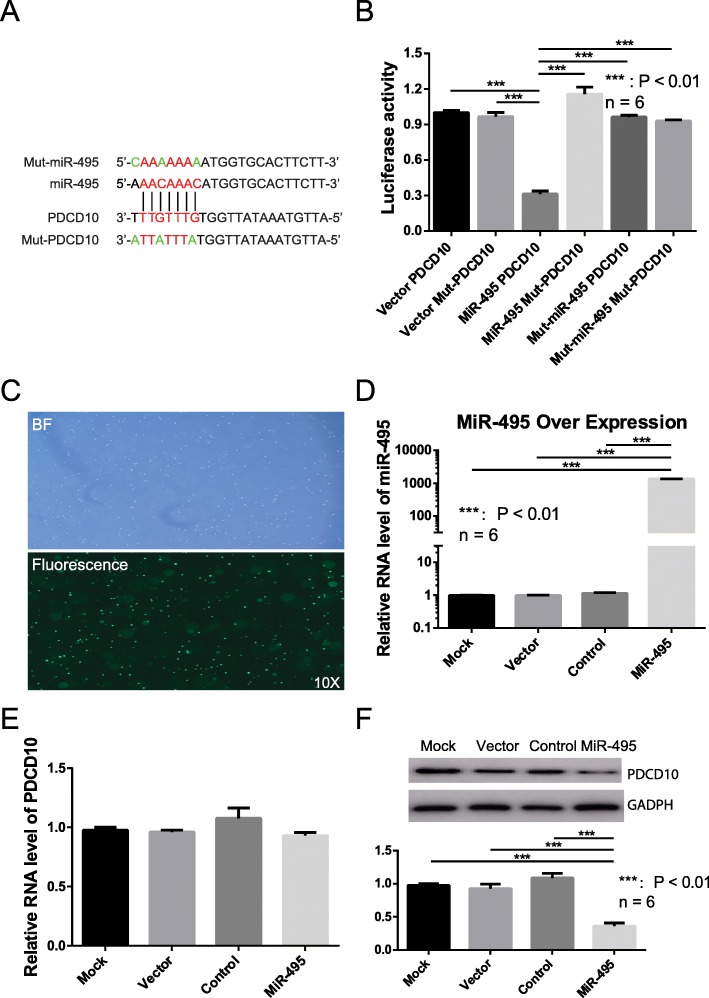
Validation the interaction between *PDCD10* and *miR-495*. **a** Schema of the interaction between *miR-495* and *PDCD10* 3′UTR. Black vertical lines and red bases indicate the interacting regions. Mutated bases are marked with green letters. **b** Dual luciferase report assay results. Vector PDCD10 and VectorMut-PDCD10 indicate plasmid with wild-type and mutated *PDCD10* 3′UTR, respectively. MiR-495 PDCD10 indicates the interaction between wild-type *miR-495* and wild-type *PDCD10* 3′UTR. MiR-495 Mut-PDCD10 indicates the interaction between wide type *miR-495* and mutated *PDCD10* 3′UTR. Mut-miR-495 PDCD10 indicates the interaction between mutated *miR-495* and wild-type *PDCD10* 3′UTR. Mut-miR-495 Mut-PDCD10 indicates the interaction between mutated *miR-495* and mutated *PDCD10* 3′UTR. **c** Transfection of primary PBMCs from AS. Top picture: bright field image of transfected PBMCs. Bottom picture: fluorescence image of transfected PBMCs. **d** Detection of *miR-495* in transfected primary PBMCs from AS. Mock: untreated group. Vector: blank vector group. Control: mutated *miR-495* over-expression group. MiR-495: *miR-495* over-expression group. **e** RNA detection of *PDCD10* in transfected primary PBMCs from AS. Mock: untreated group. Vector: blank vector group. Control: mutated *miR-495* over-expression group. MiR-495: *miR-495* over-expression group. **f** Western blot of *PDCD10* in transfected primary PBMCs from AS. Mock: untreated group. Vector: blank vector group. Control: mutated *miR-495*over-expression group. MiR-495: *miR-495* over-expression group. *n* = 6: repeated times. ***: *P* value < 0.01

In order to confirm whether *miR-495* can target *PDCD10*, in vitro tests were employed. Dual luciferase reporter assays were used to validate the target sites of *miR-495* using *miR-495* mimics, mutated *miR-495*, *PDCD10* 3’UTR, and the corresponding 3’UTR mutation (Fig. 4a); our results showed that *miR-495* mimics can strongly inhibit the *PDCD10* 3’UTR in comparison to other groups (Fig. 4b). The dual luciferase assays performed in Jurkat cells were consistent with this result (Supplementary Materials Fig. 1). Thus, the results of the dual luciferase reporter assays suggested that *miR-495* can efficiently inhibit luciferase by interacting with *PDCD10* 3’UTR.

Another in vitro experiment tested the over-expression of *miR-495* from adenovirus in primary cultures of PBMCs from patients with AS. The transfection efficiency was evaluated by the detection of *miR-495* expression and the green fluorescent protein (*GFP*) reporter gene. *GFP* and qPCR of *miR-495* showed that the transfection efficiency of the adenovirus was very high (Fig. 4c and d). The mRNA and protein expression of *PDCD10* was assessed. qPCR suggested that there was a minor effect on the *PDCD10* mRNA level (Fig. 4e), while the *PDCD10* protein expression is significantly repressed (*P* < 0.01) (Fig. 4f). *miR-495* seems to significantly inhibit the translation of *PDCD10*, while having little effect on its mRNA level.

### ROC curves of *miR-495*

**Fig. 5:**
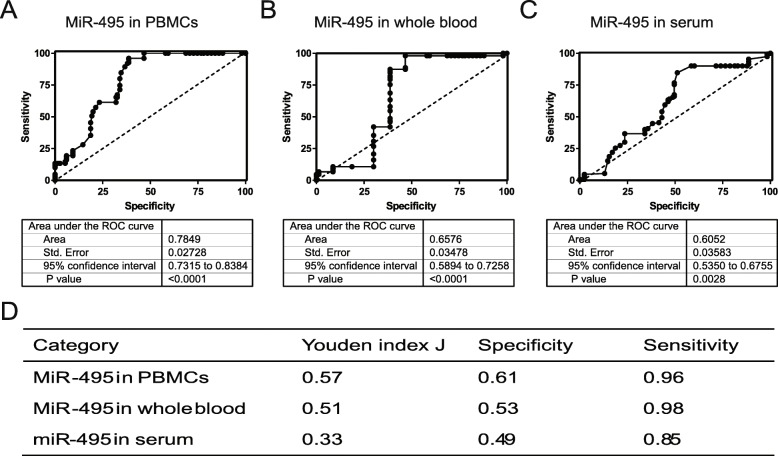
ROC curves of *miR-495*. **a** ROC curve of PBMC *miR-495*. **b** ROC curve of whole blood *miR-495*. **c** ROC curve of serum *miR-495*. **d** Summary of miR-495 specificity and sensitivity. Youden index was used to evaluate the tested methods. And the Jordan index = sensitivity + specificity-1. When the maximum value of the Jordan index was determined, the corresponding specificity and sensitivity rates of ROC curve were also achieved. PBMCs: peripheral blood mononuclear cells; ROC: receiver operating characteristic. Area, Std. Error, 95% confidence interval, *P* value, Jordan index, specificity, and sensitivity were indicated as shown

In order to evaluate the specificity and sensitivity of *miR-495* in AS diagnosis, different ROC curves of *miR-495* were plotted. The area under the ROC curve (AUC) for *miR-495* of PMBCs was 0.7849 (95% CI: from 0.7315 to 0.8384, *P* value < 0.0001) (Fig. 5a). The AUC of whole blood *miR-495* was found to be 0.6576 (95% CI: from 0.5894 to 0.7258, *P* value < 0.0001) (Fig. 5b). The AUC of serum *miR-495* was 0.6052 (95% CI: from 0.5350 to 0.6755, *P* value = 0.0028) (Fig. 5c).

The AUC values show that PBMC *miR-495* is more specific and sensitive than whole blood or serum *miR-495* for AS diagnosis. Then, the specificity and sensitivity of *miR-495* were calculated. The specificity and sensitivity of *miR-495* in the different blood components were 0.61 and 0.96, respectively, in PBMCs; 0.53 and 0.98, respectively, in whole blood; and 0.49 and 0.85, respectively, in serum (Fig. 5d). In most AS studies, miRNAs are sourced from PBMCs (Li et al. 2016; Mohammadi et al. 2018). The concentration of miRNAs in serum RNAs may be too low for efficient isolation and amplification. While a higher target concentration would be present in whole blood, this would be limited by the high level of RNA in whole blood, which may account for limited use of whole blood in AS diagnosis.

### *miR-495* can down-regulate β-catenin and TGF-β1

**Fig. 6:**
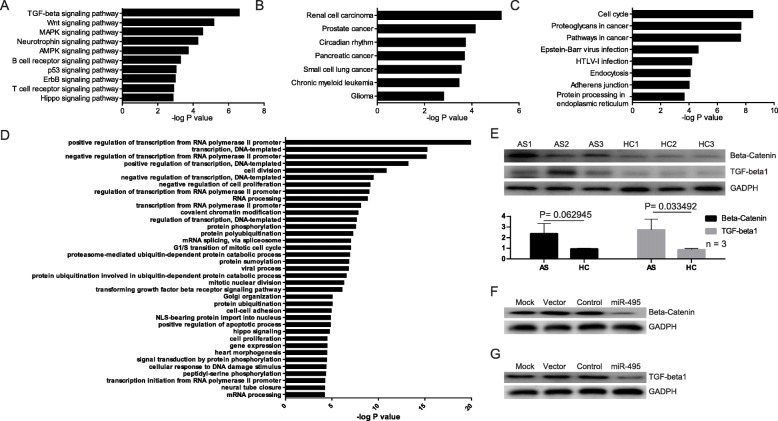
Exploring the potential role of *miR-495***. a** Classical signaling pathway analysis. **b** Signaling pathways related to disease analysis. **c** Other signaling pathway analysis. Signaling pathways were analyzed by KEGG with a *P* value threshold of 0.01. **d** GO categories of *miR-495*. The -log *P* value came from enrichment analysis. The 39 GO categories (biological processes) were all with a *P* value threshold of 0.01. **e** Western blot of β-catenin and TGF-β1. AS1, AS2, and AS3: different patients with AS. Healthy controls were indicated as HC1, HC2, and HC3. *n* = 3: repeated samples in each group. *P* values were indicated as shown. **F** Western blot of β-catenin in transfected primary PBMCs. Mock: untreated group. Vector: blank vector group. Control: mutated *miR-495* over-expression group. MiR-495: *miR-495* over-expression group. **g** Western blot of TGF-β1 in transfected primary PBMCs. Mock: untreated group. Vector: blank vector group. Control: mutated *miR-495* over-expression group. MiR-495: *miR-495* over-expression group

In order to explore the potential role of *miR-495* in AS, we used all confirmed and predicted targets of *miR-495* based on KEGG and GO analysis. Signaling pathway analysis determined that *miR-495* participates in multiple pathways (Fig. 6a, b and c). Biological processes analysis suggested that *miR-495* is versatile in gene expression regulation (Fig. 6d).

Following these analyses, the Wnt and TGF-β signal pathways were chosen for further studies. Firstly, β-catenin and TGF-β1 were detected in both AS and healthy controls, suggesting that both the related signal pathways are activated in AS (Fig. 6e). Then, the influence of *miR-495* on these same signal pathways was examined. The adenovirus containing over-expressed *miR-495* was used to infect the primary PBMCs from patients with AS. The results indicate that over-expressed *miR-495* can strongly inhibit the expression of β-catenin and TGF-β1 (Fig. 6f and g). Considering the heterogeneity of PBMCs, flow cytometry was used to assess the cell population. Our results showed that the number of lymphocytes varies between healthy controls and patients with AS (Supplementary Materials Fig. 2). Thus, we speculated that lymphocytes are more relevant for *miR-495* function in AS pathogenesis.

## Discussion

Abnormal expression of miRNAs has been deemed to be important in the pathogenesis of AS, though their exact roles are still not fully understood. Studies from Xu et al. (2015) and Qian et al. (2016) indicated that there was a strong association between AS and *miR-146a/rs2910164*, while the results from Niu et al. (2015) did not support this conclusion (Niu et al. 2015; Xu et al. 2015; Qian et al. 2016). The levels of *miR-29a* have been shown to be elevated in PBMCs (Huang et al. 2014), whereas in other studies they have been reported to be significantly lower (Lv et al. 2015). However, recent studies on AS supported an increased expression of *miR-29a* in PBMCs (Huang et al. 2019a, Yang et al. 2019b). Ma et al. (2016) found that *miR-132* is down-regulated in plasma; nevertheless, Guo et al. (2018) showed that *miR-132* is up-regulated in PBMCs (Ma et al. 2016; Guo et al. 2018). These divergent results may depend on the stages of AS, methods used, samples type (serum, plasma, PBMC, and whole blood) or the specific expression of miRNAs. In this study, our results suggested that *miR-495* is down-regulated in different biological fluids in patients with AS and the expression of *miR-495* in PBMCs is likely to be more stable than that in other blood components. These studies also suggested that miRNAs involved in AS have a more complex role than anticipated.

Previous studies on *miR-495* and its targeted mRNAs indicated it is versatile under diverse physiological and pathological conditions: its up-regulation was detected in diverse cardiomyopathies and its inhibitor (antimiR) can attenuate pathological hypertrophy (Clark et al. 2016). Up-regulated *miR-495* can induce senescence of mesenchymal stem cells derived from patients with pre-eclampsia by targeting *Bmi-1* mRNA (Li et al. 2017). In breast cancer, up-regulated *miR-495* can inhibit cell proliferation and promote apoptosis by targeting *STAT-3* (Chen et al. 2017). In osteoarthritis, up-regulated *miR-495* can promote chondrocyte apoptosis through inhibition of the NF-*k*B signaling pathway by targeting *CCL4* (Yang et al. 2019a). In this study, over-expressed *miR-495* decreased β-catenin and TGF-β1 levels, although the detailed mechanism is still unclear. Among the potential mechanisms, an alteration of the Wnt and TGF-β signal pathways seemed the most feasible, since these pathways had well-documented and essential roles in AS (Howe et al. 2005; Shehata et al. 2006; Diarra et al. 2007; Maripat.Corr 2014). Functional studies of *miR-495* could highlight its potential clinical application as a biomarker or therapeutic target in AS. We would thus like to explore all these potential interactions by high-throughput sequencing of RNAs isolated by cross-linking immunoprecipitation (HITS-CLIP), a method for decoding all microRNA-mRNA interaction maps (Chi et al. 2009; Moore et al. 2014).

Programmed cell death 10 protein is an important and versatile signal transduction regulating molecule (Huang and Zhao 2013). Under oxidative stress, *PDCD10* can affect the cellular levels of mammalian STE20-like protein kinase 4 (*MST4*) and protect cells from reactive oxygen species (Fidalgo et al. 2012). Conversely, *PDCD10* has been implicated in accelerating apoptosis via interacting with serine/threonine protein kinase 25 (*STK25*) under oxidative stress (Zhang et al. 2012). Based on previous functional studies of *miR-495*, it is reasonable to infer that *PDCD10* might promote cell growth in AS. Understanding the involvement of *PDCD10* in AS and its interactions with miRNAs is a potential avenue for further studies.

Nationwide population-based cohort studies show that patients with AS have a higher risk of developing cancer, acute coronary syndrome, Asthma, atrial fibrillation, and venous thromboembolism (Chou et al. 2014, Shen et al. 2015, Avina-Zubieta et al. 2019, Huang et al. 2019b, Moon et al. 2019). A systematic review and meta-analysis also confirms that patients with AS are at highest risk for malignancy overall (Deng et al. 2016). *miR-495* is down-regulated in multiple diseases in the experimentally supported human microRNA disease database (Huang et al. 2019b). As a potential biomarker in AS, *miR-495* showed high sensitivity but low specificity in all kinds of tissue detected. A well-studied biomarker, C-reactive protein (CRP), is a sensitive and valuable nonspecific indicator of most forms of tissue damage, inflammation, and infection, reflecting a broad range of diverse pathologic processes (Koenig and Pepys 2002). Thus, it may be desirable to favor sensitivity over specificity for the early, less expensive, noninvasive tests (Hartwell et al. 2006).

## Conclusions

In conclusion, our study confirmed that *miR-495* down-regulation could be used as a potential molecular biomarker in AS diagnosis and treatment. This study provided insights into the role of *miR-495* in AS as well as its interaction with PDCD10, but the precise mechanism of action of miR-495 and clinical applications require further experimental investigation.

## Supplementary information

**Additional file 1: Figure S1.** Dual luciferase assays in Jurkat cells. Mock: untreated group. MiR-495 PDCD10 indicated wild-type miR-495 and PDCD10 3’UTR. MiR-495 Mut-PDCD10 indicated wide type miR-495 and mutated PDCD10 3’UTR. Mut-miR-495 PDCD10 indicated mutated miR-495 and wild type PDCD10 3’UTR. Mut-miR-495 Mut-PDCD10 indicated mutated miR-495 and mutated PDCD10 3’UTR. The test was repeated six times independently (n=6). ***: *P* value < 0.01. **Figure S2.** Flow cytometry analysis of PBMCs. Fluorescent labeled anti CD3 monoclonal antibody (BD Biosciences) was combined with PBMCs to detect the expression of CD3 antigen on the lymphocytes cell surface by flow cytometer (BD Biosciences). The number of AS lymphocytes was more than the healthy controls. **Table S1.** Primers used in this study. The table was separated by 3 columns: Gene name, RT primer (From 5’ to 3’), Forward primer (From 5’ to 3’). **Table S2.** Primers used in Bisulfite-specific PCR sequencing (BSP). The table was separated by 3 columns: Gene name, Forward primer (From 5’ to 3’). **Table S3.** Primers used in Methylated DNA Immunoprecipitation (MeDIP) ChIP qPCR. The table was separated by 3 columns: Gene name, Forward primer (From 5’ to 3’).

## Data Availability

The datasets used and/or analyzed during the current study are available from the corresponding author on reasonable request.

## Abbreviations

MiRNAs: MicroRNAs
AS: Ankylosing Spondylitis
MiR-495: Hsa-miR-495-3p
qPCR: Quantitative real-time PCR
PBMCs: Peripheral Blood Mononuclear Cells
BSP: Bisulfite-specific PCR sequencing
MeDIP: methylated DNA immunoprecipitation
ROC: Receiver Operating Characteristic
PDCD10: Programmed Cell Death
GO: Gene Ontology
KEGG: Kyoto Encyclopedia of Genes and Genomes
CCMs: cerebral cavernous malformations
GCKIII: Germinal Center Kinase III
DMEM: Dulbecco’s Modified Eagle Medium
AUC: area under the ROC curve
MST4: mammalian STE20-like protein kinase 4
STK25: serine/threonine protein kinase 25

## References

[CR1] Ahmadi A, Khansarinejad B, Hosseinkhani S, Ghanei M, Mowla SJ (2017). miR-199a-5p and miR-495 target GRP78 within UPR pathway of lung cancer. Gene.

[CR2] Avina-Zubieta JA, Chan J, De Vera M, Sayre EC, Choi H, Esdaile J (2019). Risk of venous thromboembolism in ankylosing spondylitis: a general population-based study. Ann Rheum Dis.

[CR3] Bartel DP (2004). MicroRNAs: genomics, biogenesis, mechanism, and function. Cell.

[CR4] Bergametti F, Denier C, Labauge P, Arnoult M, Boetto S, Clanet M, Coubes P, Echenne B, Ibrahim R, Irthum B, Jacquet G, Lonjon M, Moreau JJ, Neau JP, Parker F, Tremoulet M, Tournier-Lasserve E, N. Societe Francaise de (2005). Mutations within the programmed cell death 10 gene cause cerebral cavernous malformations. Am J Hum Genet.

[CR5] Braun J, Sieper J (2007). Ankylosing spondylitis. Lancet.

[CR6] Brown MA, Kenna T, Wordsworth BP (2016). Genetics of ankylosing spondylitis--insights into pathogenesis. Nat Rev Rheumatol.

[CR7] Bustin SA, Benes V, Garson JA, Hellemans J, Huggett J, Kubista M, Mueller R, Nolan T, Pfaffl MW, Shipley GL, Vandesompele J, Wittwer CT (2009). The MIQE guidelines: minimum information for publication of quantitative real-time PCR experiments. Clin Chem.

[CR8] Ceccarelli DF, Laister RC, Mulligan VK, Kean MJ, Goudreault M, Scott IC, Derry WB, Chakrabartty A, Gingras AC, Sicheri F (2011). CCM3/PDCD10 heterodimerizes with germinal center kinase III (GCKIII) proteins using a mechanism analogous to CCM3 homodimerization. J Biol Chem.

[CR9] Chen Y, Luo D, Tian W, Li Z, Zhang X (2017). Demethylation of miR-495 inhibits cell proliferation, migration and promotes apoptosis by targeting STAT-3 in breast cancer. Oncol Rep.

[CR10] Chi SW, Zang JB, Mele A, Darnell RB (2009). Argonaute HITS-CLIP decodes microRNA-mRNA interaction maps. Nature.

[CR11] Chou CH, Lin MC, Peng CL, Wu YC, Sung FC, Kao CH, Liu SH (2014). A nationwide population-based retrospective cohort study: increased risk of acute coronary syndrome in patients with ankylosing spondylitis. Scand J Rheumatol.

[CR12] Clark AL, Maruyama S, Sano S, Accorsi A, Girgenrath M, Walsh K, Naya FJ (2016). miR-410 and miR-495 Are Dynamically Regulated in Diverse Cardiomyopathies and Their Inhibition Attenuates Pathological Hypertrophy. PLoS One.

[CR13] Danve A, O'Dell J (2015). The ongoing quest for biomarkers in Ankylosing spondylitis. Int J Rheum Dis.

[CR14] Dean LE, Jones GT, MacDonald AG, Downham C, Sturrock RD, Macfarlane GJ (2014). Global prevalence of ankylosing spondylitis. Rheumatology (Oxford).

[CR15] Deng C, Li W, Fei Y, Li Y, Zhang F (2016). Risk of malignancy in ankylosing spondylitis: a systematic review and meta-analysis. Sci Rep.

[CR16] Diarra D, Stolina M, Polzer K, Zwerina J, Ominsky MS, Dwyer D, Korb A, Smolen J, Hoffmann M, Scheinecker C, van der Heide D, Landewe R, Lacey D, Richards WG, Schett G (2007). Dickkopf-1 is a master regulator of joint remodeling. Nat Med.

[CR17] Eun JW, Kim HS, Shen Q, Yang HD, Kim SY, Yoon JH, Park WS, Lee JY, Nam SW (2018). MicroRNA-495-3p functions as a tumor suppressor by regulating multiple epigenetic modifiers in gastric carcinogenesis. J Pathol.

[CR18] Feldtkeller E, Khan MA, van der Heijde D, van der Linden S, Braun J (2003). Age at disease onset and diagnosis delay in HLA-B27 negative vs. positive patients with ankylosing spondylitis. Rheumatol Int.

[CR19] Fidalgo M, Guerrero A, Fraile M, Iglesias C, Pombo CM, Zalvide J (2012). Adaptor protein cerebral cavernous malformation 3 (CCM3) mediates phosphorylation of the cytoskeletal proteins ezrin/radixin/moesin by mammalian Ste20-4 to protect cells from oxidative stress. J Biol Chem.

[CR20] Griffiths-Jones S, Grocock RJ, van Dongen S, Bateman A, Enright AJ (2006). miRBase: microRNA sequences, targets and gene nomenclature. Nucleic Acids Res.

[CR21] Guo TM, Yan Y, Cao WN, Liu Q, Zhu HY, Yang L, Gao MC, Xing YL (2018). Predictive value of microRNA-132 and its target gene NAG-1 in evaluating therapeutic efficacy of non-steroidal anti-inflammatory drugs treatment in patients with ankylosing spondylitis. Clin Rheumatol.

[CR22] Hartwell L, Mankoff D, Paulovich A, Ramsey S, Swisher E (2006). Cancer biomarkers: a systems approach. Nat Biotechnol.

[CR23] Howe HS, Cheung PL, Kong KO, Badsha H, Thong BY, Leong KP, Koh ET, Lian TY, Cheng YK, Lam S, Teo D, Lau TC, Leung BP (2005). Transforming growth factor beta-1 and gene polymorphisms in oriental ankylosing spondylitis. Rheumatology (Oxford).

[CR24] Huang DN, Zhao HS. PDCD10, a Novel Signal Transduction Regulating Molecule with Multiple Functions. Chin J Biochem Mol Biol. 2013;24(9):306–10.

[CR25] Huang da W, Sherman BT, Lempicki RA (2009). Bioinformatics enrichment tools: paths toward the comprehensive functional analysis of large gene lists. Nucleic Acids Res.

[CR26] Huang da W, Sherman BT, Lempicki RA (2009). Systematic and integrative analysis of large gene lists using DAVID bioinformatics resources. Nat Protoc.

[CR27] Huang J, Song G, Yin Z, Fu Z, Zhang L (2019). Altered expression of microRNAs targeting Dkk-1 in peripheral blood mononuclear cells of patients with ankylosing spondylitis. Cent Eur J Immunol.

[CR28] Huang J, Song G, Yin Z, Luo X, Ye Z (2014). Elevated miR-29a expression is not correlated with disease activity index in PBMCs of patients with ankylosing spondylitis. Mod Rheumatol.

[CR29] Huang Z, Shi J, Gao Y, Cui C, Zhang S, Li J, Zhou Y, Cui Q (2019). HMDD v3.0: a database for experimentally supported human microRNA-disease associations. Nucleic Acids Res.

[CR30] Katial RK, Sachanandani D, Pinney C, Lieberman MM (1998). Cytokine production in cell culture by peripheral blood mononuclear cells from immunocompetent hosts. Clin Diagn Lab Immunol.

[CR31] Koenig W, Pepys MB (2002). C-reactive protein risk prediction: low specificity, high sensitivity. Ann Intern Med.

[CR32] Krol J, Loedige I, Filipowicz W (2010). The widespread regulation of microRNA biogenesis, function and decay. Nat Rev Genet.

[CR33] Lai NS, Yu HC, Chen HC, Yu CL, Huang HB, Lu MC (2013). Aberrant expression of microRNAs in T cells from patients with ankylosing spondylitis contributes to the immunopathogenesis. Clin Exp Immunol.

[CR34] Li X, Song Y, Liu D, Zhao J, Xu J, Ren J, Hu Y, Wang Z, Hou Y, Zhao G (2017). MiR-495 promotes senescence of Mesenchymal stem cells by targeting Bmi-1. Cell Physiol Biochem.

[CR35] Li Z, Wong SH, Shen J, Chan MT, Wu WK (2016). The role of MicroRNAS in Ankylosing spondylitis. Medicine (Baltimore).

[CR36] Liu C, Jian M, Qi H, Mao WZ (2019). MicroRNA 495 inhibits proliferation and metastasis and promotes apoptosis by targeting Twist1 in gastric Cancer cells. Oncol Res.

[CR37] Lv Q, Li Q, Zhang P, Jiang Y, Wang X, Wei Q, Cao S, Liao Z, Lin Z, Pan Y, Huang J, Li T, Jin O, Wu Y, Gu J (2015). Disorders of MicroRNAs in peripheral blood mononuclear cells: as novel biomarkers of Ankylosing spondylitis and provocative therapeutic targets. Biomed Res Int.

[CR38] Ma X, Wu F, Xin L, Su G, He F, Yang Y, Sun J, Liu Z (2016). Differential plasma microRNAs expression in juvenile idiopathic arthritis. Mod Rheumatol.

[CR39] Maripat.Corr (2014). Wnt signaling in ankylosing spondylitis. Clin Rheumatol.

[CR40] Mohammadi H, Hemmatzadeh M, Babaie F, Gowhari Shabgah A, Azizi G, Hosseini F, Majidi J, Baradaran B (2018). MicroRNA implications in the etiopathogenesis of ankylosing spondylitis. J Cell Physiol.

[CR41] Moon I, Choi EK, Jung JH, Han KD, Choi YJ, Park J, Cho JH, Lee E, Choe W, Lee SR, Cha MJ, Lim WH, Oh S (2019). Ankylosing spondylitis: a novel risk factor for atrial fibrillation - a nationwide population-based study. Int J Cardiol.

[CR42] Moore MJ, Zhang C, Gantman EC, Mele A, Darnell JC, Darnell RB (2014). Mapping Argonaute and conventional RNA-binding protein interactions with RNA at single-nucleotide resolution using HITS-CLIP and CIMS analysis. Nat Protoc.

[CR43] Ni W-J, Leng X-M (2015). Dynamic miRNA-mRNA paradigms: new faces of miRNAs. Biochem Biophys Rep.

[CR44] Niu Z, Wang J, Zou H, Yang C, Huang W, Jin L (2015). Common MIR146A polymorphisms in Chinese Ankylosing spondylitis subjects and controls. PLoS One.

[CR45] Qian BP, Ji ML, Qiu Y, Wang B, Yu Y, Shi W, Luo YF (2016). Identification of serum miR-146a and miR-155 as novel noninvasive complementary biomarkers for Ankylosing spondylitis. Spine (Phila Pa 1976).

[CR46] Shehata M, Schwarzmeier JD, Hilgarth M, Demirtas D, Richter D, Hubmann R, Boeck P, Leiner G, Falkenbach A (2006). Effect of combined spa-exercise therapy on circulating TGF-beta1 levels in patients with ankylosing spondylitis. Wien Klin Wochenschr.

[CR47] Shen TC, Lin CL, Wei CC, Chen CH, Tu CY, Hsia TC, Shih CM, Hsu WH, Sung FC (2015). The risk of asthma in patients with ankylosing spondylitis: a population-based cohort study. PLoS One.

[CR48] Takai D, Jones PA (2003). The CpG island searcher: a new WWW resource. Silico Biol.

[CR49] van der Linden S, Valkenburg HA, Cats A (1984). Evaluation of diagnostic criteria for ankylosing spondylitis. A proposal for modification of the New York criteria. Arthritis Rheum.

[CR50] Wang J, Chen J, Sen S (2016). MicroRNA as biomarkers and diagnostics. J Cell Physiol.

[CR51] Wang Y, Liu H, Zhang Y, Ma D (1999). cDNA cloning and expression of an apoptosis-related gene, humanTFAR15 gene. Sci China C Life Sci.

[CR52] Xu HY, Wang ZY, Chen JF, Wang TY, Wang LL, Tang LL, Lin XY, Zhang CW, Chen BC (2015). Association between ankylosing spondylitis and the miR-146a and miR-499 polymorphisms. PLoS One.

[CR53] Yang DW, Qian GB, Jiang MJ, Wang P, Wang KZ (2019). Inhibition of microRNA-495 suppresses chondrocyte apoptosis through activation of the NF-kappaB signaling pathway by regulating CCL4 in osteoarthritis. Gene Ther.

[CR54] Yang JH, Li JH, Shao P, Zhou H, Chen YQ, Qu LH (2011). starBase: a database for exploring microRNA-mRNA interaction maps from Argonaute CLIP-Seq and Degradome-Seq data. Nucleic Acids Res.

[CR55] Yang W, Yan X, Xia Q, Tao Q, Gan X, Zhang Y, Chen Z, Kong W (2019). Predisposition of six well-characterized microRNAs to syndesmophytes among Chinese patients with ankylosing spondylitis. Mod Rheumatol.

[CR56] Zhang H, Ma X, Deng X, Chen Y, Mo X, Zhang Y, Zhao H, Ma D (2012). PDCD10 interacts with STK25 to accelerate cell apoptosis under oxidative stress. Front Biosci (Landmark Ed).

[CR57] Zhang L, Hammell M, Kudlow BA, Ambros V, Han M (2009). Systematic analysis of dynamic miRNA-target interactions during C. elegans development. Development.

[CR58] Zhu W, He X, Cheng K, Zhang L, Chen D, Wang X, Qiu G, Cao X, Weng X (2019). Ankylosing spondylitis: etiology, pathogenesis, and treatments. Bone Res.

