# Supplementary material for: Down-regulated miR-495 can target programmed cell death 10 in ankylosing spondylitis
Source: Mol Med. 2020 May 25;26:50. doi: 10.1186/s10020-020-00157-3 (PMC7249445; doi:10.1186/s10020-020-00157-3)
Supplement: Supplementary file 1 — Additional file 1: Figure S1. Dual luciferase assays in Jurkat cells. Mock: untreated group. MiR-495 PDCD10 indicated wild-type miR-495 and PDCD10 3’UTR. MiR-495 Mut-PDCD10 indicated wide type miR-495 and mutated PDCD10 3’UTR. Mut-miR-495 PDCD10 indicated mutated miR-495 and wild type PDCD10 3’UTR. Mut-miR-495 Mut-PDCD10 indicated mutated miR-495 and mutated PDCD10 3’UTR. The test was repeated six times independently (n=6). ***: P value < 0.01. Figure S2. Flow cytometry analysis of PBMCs. Fluorescent labeled anti CD3 monoclonal antibody (BD Biosciences) was combined with PBMCs to detect the expression of CD3 antigen on the lymphocytes cell surface by flow cytometer (BD Biosciences). The number of AS lymphocytes was more than the healthy controls. Table S1. Primers used in this study. The table was separated by 3 columns: Gene name, RT primer (From 5’ to 3’), Forward primer (From 5’ to 3’). Table S2. Primers used in Bisulfite-specific PCR sequencing (BSP). The table was separated by 3 columns: Gene name, Forward primer (From 5’ to 3’). Table S3. Primers used in Methylated DNA Immunoprecipitation (MeDIP) ChIP qPCR. The table was separated by 3 columns: Gene name, Forward primer (From 5’ to 3’). [file 10020_2020_157_MOESM1_ESM.doc]

**Supplementary Materials**

**Fig. 1** Dual luciferase assays in Jurkat cells. Mock: untreated group. MiR-495 PDCD10 indicated wild-type miR-495 and PDCD10 3’UTR. MiR-495 Mut-PDCD10 indicated wide type miR-495 and mutated PDCD10 3’UTR. Mut-miR-495 PDCD10 indicated mutated miR-495 and wild type PDCD10 3’UTR. Mut-miR-495 Mut-PDCD10 indicated mutated miR-495 and mutated PDCD10 3’UTR. The test was repeated six times independently (n=6). ***: P value < 0.01.


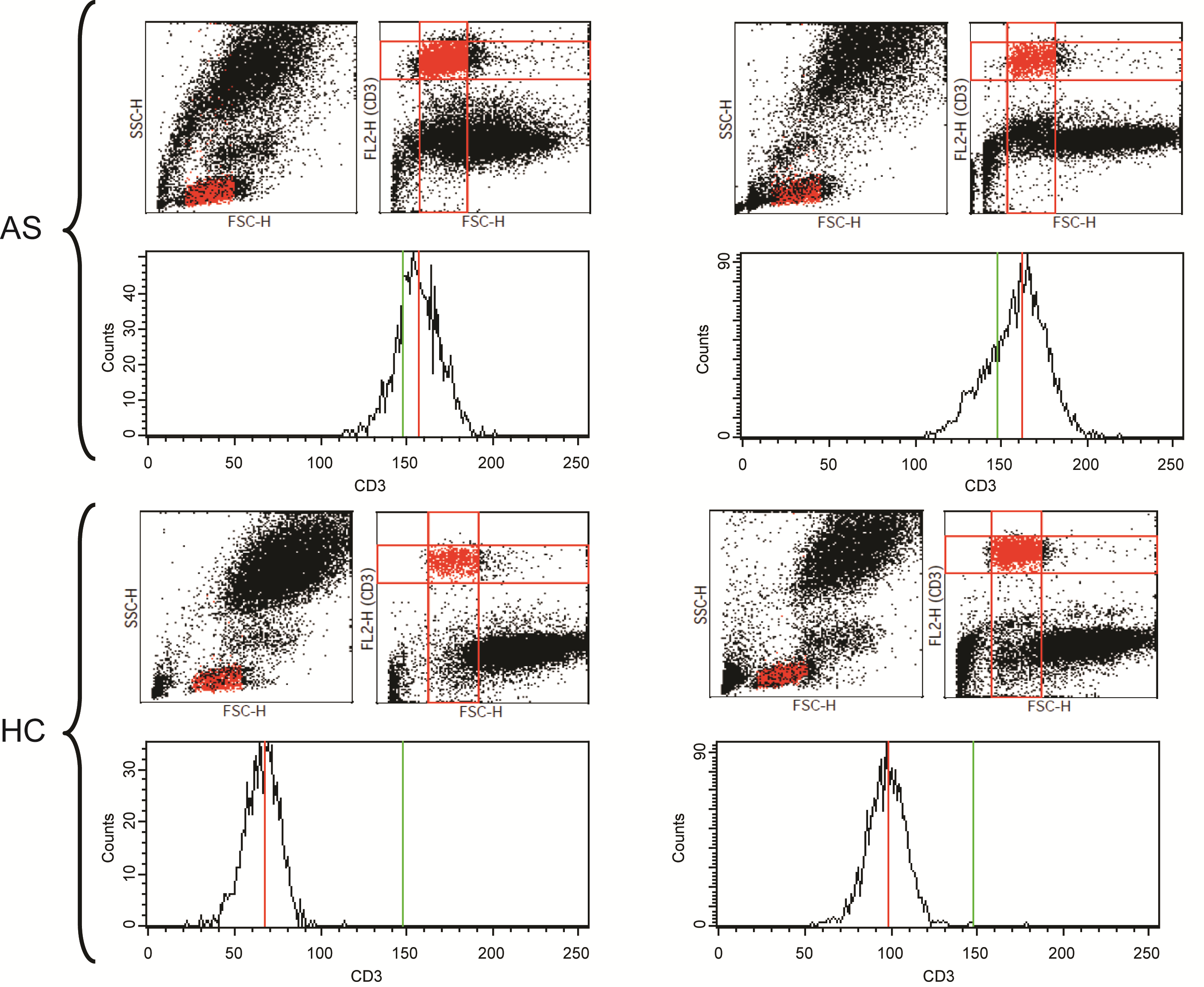


**Fig. 2** Flow cytometry analysis of PBMCs. Fluorescent labeled anti CD3 monoclonal antibody (BD Biosciences) was combined with PBMCs to detect the expression of CD3 antigen on the lymphocytes cell surface by flow cytometer (BD Biosciences). The number of AS lymphocytes was more than the healthy controls.

**S1: Table 1 Primers used in this study.** The table was separated by 3 columns: Gene name, RT primer (From 5’ to 3’), Forward primer (From 5’ to 3’).

| Names | Forward (5’ to 3’) | Reverse (5’ to 3’) |
| --- | --- | --- |
| PDCD10 | GCCCCTCTATGCAGTCATGTA | AGCCTTGATGAAAGCGGCTC |
| ACTB | GAAACTACCTTCAACTCCATC | CGAGGCCAGGATGGAGCCGCC |
| Hsa-miR-495-3p | Mimics sequences: 5’-AAACAAACAUGGUGCACUUCUU-3’ | |
| RT primer | GTCGTATCCAGTGCGTGTCGTGGAGTCGGCAATTGCACTGGATACGACAAGAAGTG | |
| PCR primers | Forward (5’ to 3’) | Reverse (5’ to 3’) |
|  | GGGCAAACAAACATGGTGCA | CAGTGCGTGTCGTGGAGT |
| Cel-miR-39 | 5’-UCACCGGGUGUAAAUCAGCUUG-3’ | |
| RT primer | GTCGTATCCAGTGCGTGTCGTGGAGTTCGCACTGGATACGACCAAGCT | |
| PCR primers | Forward (5’ to 3’) | Reverse (5’ to 3’) |
|  | GGCCTCACCGGGTGTAAATCAG | CAGTGCGTGTCGTGGAGT |

**S2: Table 2 Primers used in Bisulfite-specific PCR sequencing (BSP).** The table was separated by 3 columns: Gene name, Forward primer (From 5’ to 3’).

| Names | Forward (5’ to 3’) | Reverse (5’ to 3’) |
| --- | --- | --- |
| Hsa-miR-495-3p | CACAAGCAGCTCCAGGACAC | AAACACGGGCAACTTCTCATT |

**S3: Table 3 Primers used in Methylated DNA Immunoprecipitation (MeDIP) ChIP qPCR.** The table was separated by 3 columns: Gene name, Forward primer (From 5’ to 3’).

| Names | Forward (5’ to 3’) | Reverse (5’ to 3’) |
| --- | --- | --- |
| Hsa-miR-495-3p | AGATGTCTCCCAGGTCTTGAGG | CACCGTTGACAACACTGCTTC |
| PDCD10 | TCTCACCTCCTCCCGCAAAG | GAAGGACCCAAGAGGAACCAG |
| GADPH | ACGTAGCTCAGGCCTCAAGA | GCGGGCTCAATTTATAGAAAC |
